# The Influence of Leadership on Employees’ Work-nonwork Interface and Wellbeing: A Scoping Review

**DOI:** 10.1007/s12144-023-04762-3

**Published:** 2023-06-01

**Authors:** Jan Philipp Czakert, Rita Berger

**Affiliations:** Departament de Psicologia Social i Quantitativa, Universitat de Barcelona, Passeig de la Vall d’Hebron, 171, 08035 Barcelona, Spain

**Keywords:** Leadership, Work-nonwork interface, Wellbeing, Recovery, Review, Longitudinal

## Abstract

Many current working conditions are characterized by increasing blurred boundaries between work and nonwork with spillover that impact employees’ and recovery processes and wellbeing. Research, although emerging, considers these processes in the leadership-wellbeing relationship insufficiently. The main aim of this study, therefore, was to enhance our understanding of the role of leadership on employee’s work-nonwork interface and wellbeing. To address these processes adequately, longitudinal research is most appropriate. To our best knowledge, no review exists that could inform longitudinal studies on the leadership-employee wellbeing relationship with a focus on spillover and recovery processes. Following the PRISMA Extension for Scoping Reviews, we apply a narrative synthesis of 21 identified studies to organize the research landscape. We make three main contributions: First, we adopt an integrated resource-demands based process perspective and expand the leadership-employee wellbeing relationship by including spillover and recovery. Second, we map the used theoretical approaches and analyzed research gaps. Third, we offer a list of the issues and potential remedies of applied methodologies to orient further research. Results show, that while work-nonwork research is predominantly approached from a negative conflict-based view, research focused more on positive than on negative leadership. We identify two broad categories of investigated mechanisms, namely bolstering/hampering mechanisms, and buffering/strengthening mechanisms. Findings also highlight the importance of personal energy resources and therefore call for more attention to affect-driven theories. The identified predominance of the IT and healthcare sectors and of working parents warrants more representative research. We offer recommendations to advance future research both theoretically and methodologically.

## Introduction

The Sustainable Development Goals 3 (Health and well-being) and 8 (Decent work and economic growth) of the United Nations (United Nations, 2015) and the reports of the European Agency for Safety and Health at Work (2022a, b) reflect the growing importance and attention to employee wellbeing (EWB) – feeling happy and/or fulfilled during work (Sonnentag, 2015) – as a fundamental human aspiration, an increasing societal concern, and a basis for policymaking. Despite its relevance, recent reports and research suggest that EWB is at significant risk and that bolstering EWB or buffering illbeing is becoming an increasingly difficult leadership task (Adecco, 2022; Kniffin et al., 2021; Rudolph et al., 2021). Particularly, engaging in important off-work recovery processes crucial to EWB seem more challenging for many employees (Adecco, 2022; Sonnentag et al., 2022). This effect has been amplified by the emergence of more flexible and remote work contexts (McKinsey, 2021) and an increased blurring of the boundaries between work and nonwork (Cham et al., 2021; Sonnentag et al., 2022).

Moreover, although decades of leadership research have demonstrated that both positive leadership (referring to favorable scores on all sorts of leadership behavior instruments) and negative leadership (its antithesis) have a significant impact on EWB, especially in times of crisis (Rudolph et al., 2021), the considerable number of nonsignificant results found for leadership-EWB interventions indicate that more research needs to be done in this area (Nielsen & Taris, 2019). However, from a leadership research and organizational perspective, EWB has been studied primarily because of its critical importance to organizational interests, i.e., to increase job performance (e.g., Diener et al., 2020; Nielsen & Taris, 2019). Although the research about the leadership-EWB relationship has increased over the past few years (Arnold, 2017; Harms et al., 2017; Inceoglu et al., 2018; Montano et al., 2017; Yao et al., 2021), the predominant focus on leadership-performance relationships has treated EWB rather as a secondary outcome variable, resulting in a narrow-focused understanding of EWB (Inceoglu et al., 2018; Montano et al., 2017). Specifically, whereas the research field on EWB has already evolved towards a more holistic work-nonwork perspective, including spillover and recovery processes (Inceoglu et al., 2018; Nielsen & Taris, 2019; Sonnentag et al., 2022), this work-nonwork perspective has yet to be widely applied in the leadership-EWB research. Until now, work domain-specific relationships between leadership behavior and performance-related aspects remain predominant. As a result, leaders’ potential influence on employee’s work-nonwork interface, including spillover and recovery processes, still remains a black box.Yet, there is widespread agreement that incorporating recovery and spillover processes in the study of the leadership-EWB relationship is crucial for improving our understanding of EWB's evolution over time (Bakker & De Vries, 2021). To do so, a focus on longitudinal leadership-EWB research is thus needed, as they best capture the dynamic short- and long-term processes underlying EWB (Sonnentag et al., 2022).

Generally, surprisingly little effort has been undertaken to organize longitudinal leadership-employee outcome research (Kelemen et al., 2020). To the best of our knowledge, no leadership-wellbeing review exists that focuses specifically on the work-nonwork area with spillover and recovery-related longitudinal research. However, a consequent mapping of the related theoretical approaches, used concepts, and investigated mechanisms is highly warranted to open the leadership-wellbeing research for the current context and to orient future longitudinal studies in this complex field conceptually. Additionally, intensive longitudinal studies are complex and would benefit from methodological orientation in the form of an overview of prevalent methodological issues and potential remedies. Regarding practical implications, it is necessary for appropriate diagnosis, interventions and policymaking to provide new insights for leaders and Human Resources that help to address recovery and spillover processes in design and training for bolstering EWB – and buffering illbeing respectively. Therefore, it is necessary to improve the understanding linkages between work and nonwork processes.

To fill this lacuna, the general objective of this study was to enhance our understanding of spillover and recovery processes within the leadership-EWB relationship by means of a qualitative scoping review focusing on longitudinal studies. We want to answer four questions: First, which investigated theoretical approaches and concepts can be identified in this particular field? Second, what categories of mechanisms under study can be identified? Third, which theoretical issues can be identified? Fourth, which methodological issues and potential remedies can be identified?

The scoping review contributes to the leadership-EWB literature in three main ways.

Firstly, by adopting an integrated resource-demands based process perspective, we include the significant role of spillover and recovery processes and expand and organize the leadership-EWB literature accordingly. In doing so, we open avenues for leadership-EWB research for the investigation of resources and demands across the work-nonwork interface, implicating that leaders can also influence spillover and off-work recovery processes. We also demonstrate that related research is too reliant on leadership theories that are rooted in leadership-performance relationships, whereas research would benefit from EWB-grounded theories (Russell, 1980; Weiss & Cropanzano, 1996) as a starting point for theory development.

Secondly, by categorizing the used concepts and mechanisms in the leadership-EWB relationship across the work-nonwork interface, we inform future research about the limitations of used theories and mechanisms and highlight the areas that need further development. We identify a wide range of different applied theories while showing that a coherent integral theory is lacking. And while positive psychology approaches, related theories, and concepts (positive spillover, positive EWB) all need more attention, negative leadership behaviors and styles – especially absent leadership – also warrant more investigation. We also discuss issues of broad leadership conceptualizations versus specific behaviors. Regarding modelling options, the main takeaway is the identification of two main categories of mechanisms that may be challenged or tested in future studies: Based on positive psychology (e.g., Waters et al., 2022), we propose to differentiate leadership-EWB relationships between bolstering/hampering mechanisms (i.e., leadership as a predictor) and buffering/strengthening mechanisms (i.e., leadership as a moderator).

Thirdly, methodological issues of the screened papers are highlighted, and potential remedies are discussed to advance future research. The review resulted in articles including a vast array of different methodological designs, including randomized field trials, multiple wave studies, and mainly diary studies. We identify methodological limitations of these screened studies originating in design, results, and external validity, and highlight potential remedies. Finally, we offer recommendations for related future research both theoretically and methodologically to push the knowledge frontiers of this research field further.

We structure our review in four main sections: the first section underlines the theory we used for our review; next, we describe the methodology that we applied to search and code papers; the third section identifies the theoretical approaches, categorizes the researched mechanisms, and presents the findings on theoretical and methodological issues. The fourth section discusses the findings in light of the theory and suggests implications for scholars, practitioners, and policymaking.

## Underpinning theory

To integrate work-nonwork research including spillover and recovery processes into the leadership-EWB relationship, we add a dynamic process perspective to the relatively static resource-demand perspective.

Resource-demands perspectives based on Hobfoll’s (1989) conservation of resources theory and occupational psychology derivates such as the job demands-resource model (Bakker & Demerouti, 2007) have been widely used in EWB (Inceoglu et al., 2018) and leadership research (e.g., Schaufeli, 2015; Kelemen et al., 2020), and have been successfully adapted for work-nonwork interface research through the work-home resources model (ten Brummelhuis & Bakker, 2012). The resource-demand-based perspective generally distinguishes between resources, i.e., aspects that potentially help individuals to maintain their wellbeing, on the one hand, and demands, i.e., aspects that potentially impair wellbeing, on the other hand (Berger et al., 2019; Berger & Czakert, 2022; Lesener et al., 2019; Quinn et al., 2012). This perspective explains how interactions of these factors result in either wellbeing or illbeing. Resources and demands may be categorized into contextual (e.g., working conditions) and personal (e.g., human energy) factors (ten Brummelhuis & Bakker, 2012).

The work-nonwork interface adds spillover and recovery processes to the distinction of resources and demands and can be referred to as “the interaction of employee work experiences and [nonwork] lives” (Allen, 2012, p. 1163), where both negative (i.e., conflict or strain-based) and positive (i.e., enriching) spillover and recovery processes can happen in various forms (e.g., Bowling et al., 2010; Geurts et al., 2005; Hanson et al., 2006). Spillover theory has a long history in work-nonwork and EWB research (Bowling et al., 2010; Edwards & Rothbard, 2000), is based on role theory (Kahn et al., 1964), and assumes that experiences in one domain, e.g., the work domain, influence experiences in the other domain, e.g., the nonwork domain. Regardless of the spillover quality (i.e., positive, representing a resource, or negative, representing a demand), one of our key assumptions is that increasingly blurred boundaries between work and nonwork due to digitalization and flexibilization increase the probability of spillover processes occurring (Cham et al., 2021; Sonnentag et al., 2022). This means that, for example, psychophysiological load reactions that result from encountering work-related demands could affect more easily important recovery processes and consequently EWB (e.g., Bennett et al., 2018; Parker et al., 2021; Sonnentag & Schiffner, 2018; Sonnentag et al., 2022). Spillover processes from work to nonwork may thus be seen as the linking pin between demands and resources experienced at work and related recovery processes off-work (Edwards & Rothbard, 2000). Daily recovery processes in turn are crucial to restoring resource losses, e.g., in the form of experienced psychophysiological energy depletion during work time (Meijman & Mulder, 1998; Sonnentag, 2015). Previous meta-analyses have highlighted the importance of recovery processes for EWB (Bennett et al., 2018; Steed et al., 2021). The meta-analysis by Bennett et al. (2018) showed that the related job demands-resources-recovery model (JD-R-R) (Kinnunen et al., 2011) explains EWB better than models that do not take recovery processes into account. We thus expand the JD-R-R model by adding leadership and spillover processes to the equation.

Specifically, our review focuses on leadership behaviors as they are more closely related to spillover, recovery processes, and EWB as proximal outcomes of leadership behaviors than leadership characteristics (Inceoglu et al., 2018). We understand leadership behavior as an influencing process (Antonakis & Day, 2017; Schippers & Hogenes, 2011; Yukl, 2013) and as a core contextual concept for EWB by influencing employees’ perception of personal and contextual resources and demands. In this sense, leadership behavior can be seen either as a contextual resource (e.g., forms of positive leadership behaviors) or a contextual demand (e.g., forms of negative or absent leadership behaviors) (e.g., Berger et al., 2019; Schaufeli, 2015). As such, we assume that the way leaders may influence EWB underlies two distinct mechanisms. Firstly, leaders may shape both, resources and demands, and thereby bolster or hamper EWB (which we later refer to as bolstering/hampering mechanisms). Notably, boundary conditions for leadership apply here, depending on the leaders’ role capacity to change working conditions (Bakker & de Vries, 2021; Nielsen & Taris, 2019). Secondly, a leader may be seen as a resource or demand itself that buffers or strengthens stressor-strain relationships (which we later refer to as buffering/strengthening mechanisms). To further organize the literature, we use a wide approach and classify both specific leadership behaviors and more broad styles as positive or negative, or absence of behavior, including task and relationship orientation (Gurt et al, 2011; Kelloway & Gilbert, 2017). The categories of positive (i.e., leadership as a resource), negative (i.e., leadership as a demand), absence of leadership (i.e., leadership as a demand) (Aasland et al., 2010; Wang et al., 2021) have shown to impact EWB differentially (Montano et al., 2017). The related distinction between task-related and relationship-related leadership support has also been applied in leadership-EWB research (e.g., Yao et al., 2021).

We conceptualize EWB as a function of demands, resources, spillover and recovery processes. This perspective allows us to understand EWB as a continuum ranging from acute positive feelings of pleasure and/or fulfillment that occur in a single workday to more persistent and sustained forms of such experiences, where spillover and recovery processes influence the occurrence and persistence of these processes. We thus understand EWB as an individual-level multidimensional concept (Arnold, 2017) that is dynamic in nature: Dynamic EWB may be defined as a desirable state of “feeling good and/or experiencing fulfillment and purpose” (Sonnentag, 2015, p. 262) related to work, thus consisting of affective wellbeing (i.e., feeling good) and psychological wellbeing (i.e., experiencing fulfillment and purpose) elements. Dynamic wellbeing can fluctuate over time (Sonnentag, 2015). Accordingly, sustainable EWB may then represent dynamic EWB that can be sustained for a period of time (Di Fabio, 2017).

## Method

### A primer on the choice for conducting a scoping review

The present study carries out a scoping review of longitudinal research on the leadership-work-nonwork interface-EWB relationships to examine how research is conducted on this specific field. Specifically, we aimed to identify key theoretical approaches and concepts, the mechanisms that have been applied as well as any methodological issues (Munn et al., 2018). We are of the opinion that the current types and forms of evidence valuable to practitioners in the field of leadership and EWB needs further expanding (Arksey & O'Malley, 2005; Munn et al., 2018). Since our primary aim is to identify, map, and discuss concepts, a scoping review is the most suitable evidence synthesis approach (Munn et al., 2018), because they are particularly effective at identifying clear knowledge gaps. Scoping reviews have also proven to be highly effective at highlighting predominant methods (e.g., Callary et al., 2015), which was also of primary interest. Scoping reviews are only slightly different from systematic reviews in the following aspects: 1) Prior registration of the review protocol is not required; 2) critical appraisal is not mandatory; and 3) a generation of quantitative “summary findings” is not aim of the study (Munn et al., 2018).

### Scoping review procedure

The scoping review was based on the Preferred Reporting Items for Systematic Review and Meta-Analysis Extension for Scoping Reviews (PRISMA-ScR; Tricco et al., 2018) to ensure methodological and reporting quality. To avoid potential research duplication, the PROSPERO International Prospective Register of Systematic Reviews database had been preliminary searched for similar already undergoing reviews. No registered review matched the present study objectives, so the review process was continued.

Following guidance on the conduct of narrative synthesis in systematic reviews by Popay et al. (2006) and Siddaway et al. (2019), the individual research questions formed the basis of a refined search strategy. To ensure standardized data collection, a standardized data abstraction form was created by the first author to determine which data to extract for this specific study, the second author revised and agreed on this form. This data abstraction form was based on the research objectives and summarizes information regarding author, year, study design, sample, sample size, leadership style/behavior/theory, main findings, and main limitations of the study. Leadership behavior was coded as positive or negative. EWB was coded as positive or negative EWB, affective, psychological, or combinations of both. The third concept, work-nonwork interface, was coded as positive spillover versus negative spillover approaches, and recovery. The mechanisms were coded as bolstering/hampering mechanisms versus buffering/strengthening mechanisms taking into account the theoretical and methodological positioning of leadership, being a predictor (bolstering/hampering) or moderator (buffering/strengthening) in the leadership-EWB relationship.

We grouped the articles into two broad categories, based on their design. Studies in category 1 deployed a long-term study design, i.e., two or three wave design, including group-randomized field trials. Studies in category 2 deployed the experience sampling method (ESM) to account for short-term relationships with EWB.

### Literature search and selection

The literature search was conducted independently by two reviewers in February 2021: MEDLINE, CINAHL, PsycInfo, and PsycArticles databases were screened using the EBSCOhost research platform and special sections of COVID-19 research on researchgate.net and of journals for empirical peer-reviewed studies published between 2001–2021 in English. Conceptual dissertations, abstracts, books, and unpublished studies were excluded. Theoretical studies were excluded from this review. Cross-sectional studies, case series, and case reports were also excluded.

**Fig. 1:**
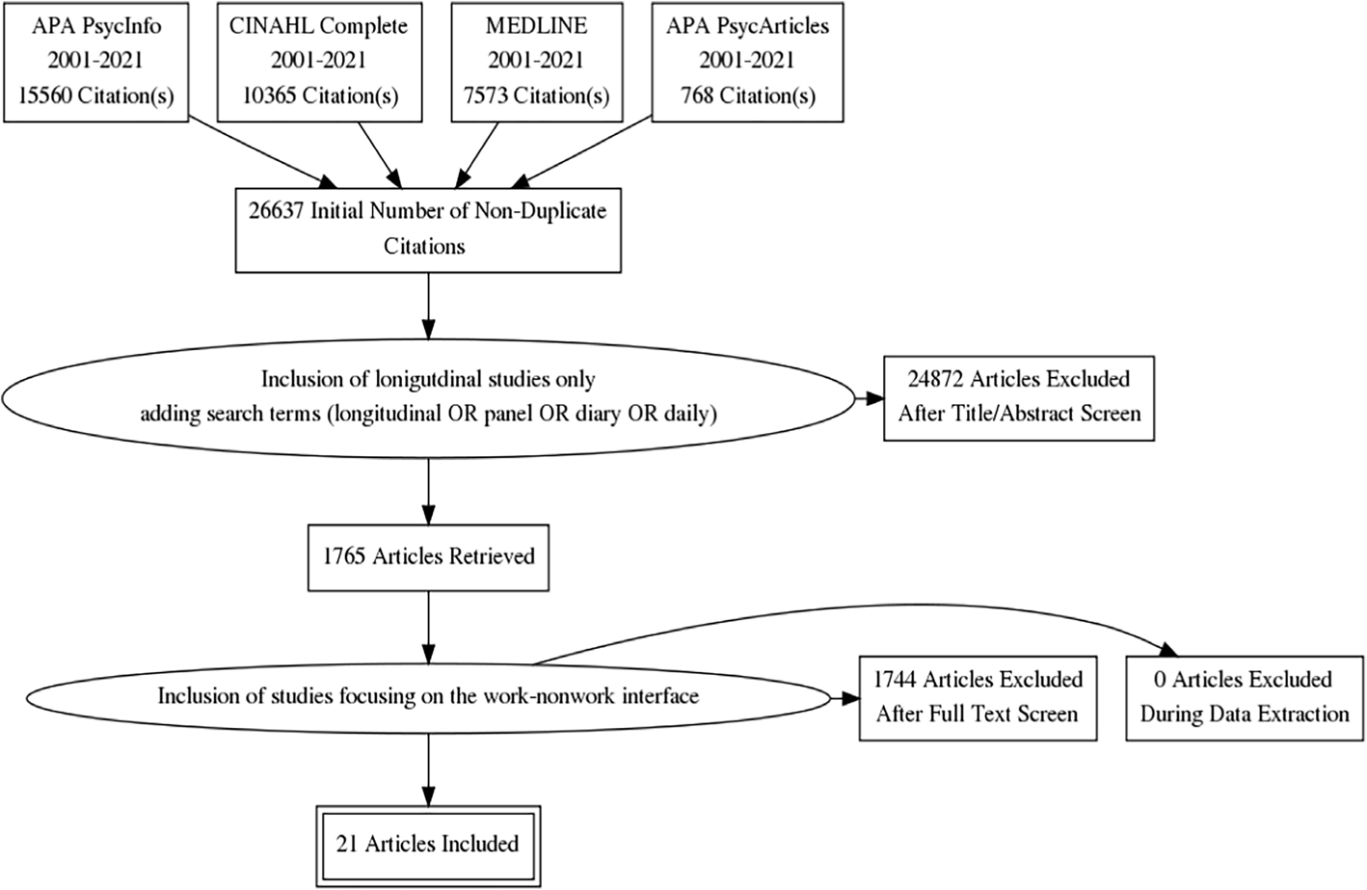
PRISMA study flow chart

The search strategy followed a multi-step procedure, in which further criteria were subsequently added after each step (see also Montano et al., 2017). The main terms of the initial search were “leadership” and “wellbeing”; related terms were defined through thesaurus browsing and were combined with the appropriate Boolean operators AND/OR (see Annex Table 6 for complete search terms and strings). We applied search strings that included related terms from Montano et al. (2017), including terms such as e.g., “transform* leader*” or “health-oriented leader*”, “positive affect”, “health issues”. This search yielded 26.637 articles. Since this review focused on longitudinal findings, we added the terms “longitudinal or panel or diary or daily*” to refine the search, resulting in 1.765 articles (= 6.6%). These articles were screened by title and abstract for the inclusion of spillover and recovery processes, represented in terms based on Beigi et al. (2019), who provided a taxonomy of work-nonwork-related constructs, and included terms such as e.g., “work-nonwork interface”, “work-nonwork spillover”, or “work–home interface”. A hand search of the reference lists in each of the retrieved papers was performed to find further potentially eligible papers. However, all additionally screened papers that addressed the topic of leadership as a predictor of EWB did not account for the work-nonwork interface (e.g., Xanthopoulou et al., 2012), and papers that addressed dynamic EWB did not specifically address leadership as antecedent (e.g., Peiró et al., 2019). Finally, the selection process yielded a final number of 21 articles, with almost perfect agreement between the two reviewers (Cohen’s κ = 0.99) (Landis & Koch, 1977). All discrepancies were resolved by a third reviewer. Figure 1 illustrates the study flow of the review search and selection process.

To identify methodological issues, and to evaluate the quality of reported evidence in a systematic way, the Grading of Recommendations Assessment, Development and Evaluation (GRADE) approach (Guyatt et al., 2008) was used, as this approach ensures a well-established transparent and simple quality appraisal. Since the GRADE system is predominantly used in clinical research – Cochrane reviews –, reasons for grading were adapted to the present research aim. Specifically, the quality of each paper was assessed in duplicate analyzing method (e.g., sampling, temporal lenses), results (e.g., effect sizes, potential confounding effects) and limitation parts. Following GRADE, the following four classifications for quality of evidence were used: High quality (further research is very unlikely to change our confidence in the estimate of effect); Moderate quality (further research is likely to have an important impact on our confidence in the estimate of effect and may change the estimate); Low quality (further research is very likely to have an important impact on our confidence in the estimate of effect and is likely to change the estimate); Very low quality (any estimate of effect is very uncertain).

## Results

In Table 1 and 2, we provide a summary of all the articles identified for our review. To begin with, while screening publications within the period of 2001 – 2021, our literature review provides evidence that researching endeavors regarding leadership as contextual variable for EWB longitudinally have only begun about ten years ago (e.g., Hornung et al., 2011). As expected, the number of identified research papers was rather small with *N* = 21. It is worth noting that more than half of our screened articles (= 52.4%) were published between 2018–2021. This demonstrates that there is increased interest in this research topic.

**Table 1:** Category 1 articles identified for review and summaries

Nr	Reference (Year)	Method			Theoretical approach		Key findings
		Sample	Sample size	Study design (Data collection duration & frequency)	Leadership Influence	Overall framework	
1	Davis et al. (2015)	IT workers	93	1 year (2-wave)*	Family Supportive Supervisor Behavior	Work-home resources model	Intervention (including FSSB) leads to significant increase in parent–child shared time
2	Demerouti et al. (2013)	Working parents in Japan	471	1 year (2-wave)	Support	Role theory	Supervisor support leads to work-self facilitation. Work-self facilitation leads to decreased psychological distress and increased happiness
3	Fan et al. (2019)	IT workers	1610	18 months (2-wave)	Family Supportive Supervisor Behavior	Job Demands Resource Model (JDR)	FSSB leads to increased decision authority (job strain resource) and increased schedule control (time strain resource), increased job satisfaction and decreased emotional exhaustion
4	Hornung et al. (2011)	German hospital physicians	159	1 year (2-wave)	Leader consideration	Employee centered production centered	Leader consideration leads to increased development and flexibility idiosyncratic deals (i-deals). Development i-deals related positively to work engagement, flexibility i-deals related negatively to work-family conflict
5	Liang et al. (2018)	Full-time employees	168	1 year (2-wave)	Abusive Supervision	Self-determination theory (SDT)	Abusive supervision leads to somatic complaints via ruminative thinking about work
6	Liu et al. (2021)	Nurses	266	9 weeks (3-wave)*	Meaningfulness communication	Transactional Model of stress; Event system theory	Interventions (including leader communication) significantly decreased perceived COVID-19 crisis strength and increased work meaningfulness. Work meaningfulness moderates the negative impact of COVID-19 on an employee’s work engagement and taking charge at work
7	Moen et al. (2016)	IT workers	867	1 year (3-wave)*	Family Supportive Supervisor Behavior	Job Demand Control Model (JDC)	FSSB does not mediate the effects of the intervention to reduced work-family-conflict nor reduced burnout, nor reduced psychological distress
8	Munir et al. (2012)	Danish elderly care workers	188	18 months (2-wave)	Transformational Leadership	Transformational leadership theory	Transformational leadership leads to decreased work–life conflict, increased job satisfaction and increased psychological wellbeing. Work–life conflict mediated between transformational leadership and wellbeing, but not job satisfaction
9	Stocker et al. (2019)	Swiss employees	208	13.8 months (2-wave)	Appreciative leadership	Stress-as-offense-to-self (SOS) theory	Appreciation by supervisors moderated the effects of interruptions on the four parameters of employees’ well-being: job satisfaction, self-efficacy, job-related depressive mood, and sleep problems

^*^ Group-randomized field trial

**Table 2:** Category 2 Articles identified for review and summaries

Index-Nr	Reference (Year)	Method			Theoretical approach		Key findings
		Sample	Sample size	Study design	Leadership Influence	Overall framework	
1	Barnes et al. (2020)	Italian employees	127	Diary 10 Days (1 MP)	Leader sleep devaluation	Abusive Supervision; Self-regulation; Social learning theory (SLT)	Sleep devaluing leader behavior leads to decreased sleep quality, which in turn leads to increased unethical behavior
2	Blanco-Donoso et al. (2017)	Spanish Nurses	74	Diary 5 Days (2 MP)	Supervisor support	Conservation of resources (COR) theory	Coworker support -but not supervisor support -, psychological detachment and relaxation minimize the unfavorable effects on well-being of difficulties in emotion regulation
3	Breevaart and Bakker (2018)	Dutch teachers	271	Diary 10 Days (1 MP)	Transform-ational leadership behavior	Job demands–resources (JD-R) theory	Daily transformational leadership behavior does not buffer the hindering effect of family-work conflict on employees’ engagement; Daily transformational leadership moderates the paths between daily challenging demands as well as daily hindrance demands on work engagement
4	Breevaart and Tims (2019)	Dutch teachers	271	Diary 10 Days (1 MP)	Supervisor support	Conservation of resources (COR) theory	Supervisor support is particularly important on days when exhaustion is high, and is crafted more often followers perceive low (vs. high) job insecurity
5	Cangiano et al. (2019)	Full-time employees	94	Diary (5–7 Days, 3 MP)	Punitive Supervision	Self‐determination theory (SDT); Stressor‐detachment model	Punitive supervision moderates the daily negative effects of proactivity on end‐of‐workday anxiety, and hence bedtime detachment
6	Chong et al. (2020)	Full-time teleworkers	120	Diary 10 Days (1 MP)	Organizational support	Conservation of resources (COR) theory	Higher (vs. lower) telework task support moderates the positive relation between end-of-day exhaustion and next-day work withdrawal behavior
7	Derks et al. (2015)	Full-time employees	100	Diary 4 Days (1 MP)	Social norm expectations	Boundary theory; Role model theory	Supervisors' expectations regarding smartphone availability in private hours moderate the link between off-work smartphone use and daily work-home interference
8	Liu et al. (2015)	Chinese IT workers	125	Diary 3 Weeks (4 MP)	Perceived managerial family support	Social exchange theory (SET); Self-regulation perspective	Perceived managerial family support moderated the impact of morning family-to-work conflict on afternoon emotional exhaustion
9	Rodríguez-Carvajal et al. (2019)	Spanish Full-time employees	122	Diary 5 Days (3 MP)	Servant leadership	Self-determination theory (SDT); Servant leadership theory	Servant leaders’ behaviors leads to increased meaning in life and vitality, which in turn, lead to increased work goal attainment
10	Stocker et al. (2014)	Swiss employees	139	Diary 5 Days (2 MP)	Appreciative Leadership	Stress-as-offense-to-self (SOS) theory; Effort-Reward imbalance	Daily appreciation by the supervisor leads to serenity at the end of workday. Appreciation by supervisors did not lead to well-being more strongly than appreciation from other sources
11	Syrek and Antoni (2014)	German IT workers	135	Diary 5 Days (2 MP)	Leader performance expectations	Conservation of resources (COR) theory; Effort-Recovery theory (ERT)	Leader performance expectations moderates the relationship between unfinished tasks and both rumination and sleep (also over the weekend)
12	Wang et al. (2019)	Employees	58	Diary 10 Days (1 MP)	Interpersonal justice	Interpersonal justice; Recovery	Variability in interpersonal justice explained unique variance in psychological detachment beyond the average level of interpersonal justice. Perceived supervisor-related interpersonal justice leads to psychological detachment, which in turn leads to positive and negative affect

*Note. MP* = Measurement points per day

### Theoretical approaches

Table 3 summarizes the theoretical approaches identified in the reviewed papers. In the following, to answer the research question “Which investigated theoretical approaches and concepts can be identified in this particular field?”, we describe the identified theoretical approaches for these three research streams (leadership, work-nonwork interface, EWB) to disentangle the complex theory development of this research stream.

**Table 3:** Theoretical approaches identified in the screened articles

Theories	Number of studies
Stressor/strain theories	17
Situational perspectives	10
Conservation of resources (COR) theory	4
Job demands–resources (JD-R) theory	2
Job Demand Control Model (JDC)	1
Work-Home Resources Model (WH-R)	1
Transactional Model of stress	1
Event system theory	1
Regulation perspectives	7
Effort-Recovery theory	2
Stressor‐detachment model	1
General recovery theory	1
Stress-as-offense-to-self (SOS) theory	2
Self-regulation theory	1
Motivational theories	3
Self-determination theory (SDT)	3
Support theories	8
General organizational and supervisor support	4
Family supportive supervisor behavior	3
Managerial family support	1
Leadership theories	9
Transformational leadership	2
Appreciative leadership	2
Servant leadership	1
Abusive supervision	2
Punitive supervision	1
Leader consideration	1
Social theories	3
Social exchange theory	2
Social learning theory	1
Justice related theories	2
Effort-reward imbalance	1
Interpersonal justice	1
Role theories	2
Role conflict theory	1
Boundary theory	1
Affect-driven theories	2
Affective/emotional circumplex	2

Number of theories do not coincide with number of articles, because several articles applied multiple theories. Specific leadership behaviors and social norm expectations were excluded for reasons of parsimony

#### Leadership theoretical approaches

As for leadership frameworks, we broadly distinguished positive, negative, and absence leadership styles/behaviors/theories. 71% of the papers (n = 15) focused on positive leadership styles/behaviors/theories (i.e., leadership as a resource), and 24% (*n* = 5) investigated the influence of negative leadership styles/behaviors/theories (i.e., leadership as a demand). One paper investigated the influence of both positive and negative leadership behaviors. None of the papers considered absence leadership styles/behaviors/theories.

Positive leadership styles/behaviors/theories included general and more work-nonwork-related specific forms of support. Studies researching about general support included general organizational and supervisor support (*n* = 4). Research on more specific support analyses include perceived managerial family support (n = 1) or family supportive supervisor behavior (FSSB; Crain & Stevens, 2018; Hammer et al., 2009) (*n* = 3). Specific behaviors focused solely on relationship-related support (*n* = 2) and included meaningful communication (*n* = 1) and appreciative behaviors (*n* = 1). Additionally, health-related concepts of appreciative (*n* = 2) and servant (*n* = 1) leadership were applied. Transformational (*n* = 2) leadership was the only performance-based style.

Negative leadership styles/theories include relationship-related orientations such as abusive supervision (*n* = 2) and punitive supervision (*n* = 1). Specific negative leadership behaviors referred to leader’s sleep devaluation, adverse performance expectations (*n* = 2), and shifts in interpersonal justice behaviors (*n* = 1).

Besides this, we observed a wide array of broader leadership-related theories used to describe the influence of leadership on EWB. This includes social theories (*n* = 3) such as social exchange theory (*n* = 2) (Cropanzano & Mitchell, 2005) and social learning theory (*n* = 1) (Bandura, 1977, 1985), justice related theories (*n* = 2) such as effort-reward imbalance (*n* = 1) (Siegrist, 2002) and interpersonal justice (*n* = 1) (Colquitt, 2001), as well as the historical distinction between “initiating structure” versus “consideration” (Kelloway & Gilbert, 2017).

#### Work-nonwork interface theoretical approaches

We distinguished approaches based on positive (*n* = 5) and negative (*n* = 16) spillover approaches (Beigi et al., 2019). Most papers identified in our review investigated forms of negative spillover. Negative spillover research studied broader (*n* = 14/16) and more narrow forms of work-nonwork conflicts (*n* = 2/16). Reverse family-to-work conflict was only investigated by a single paper (Liu et al., 2015). Positive spillover was studied, e.g., in forms of optimized time allocation (*n* = 3) (Davis et al., 2015; Fan et al., 2019; Hornung et al., 2011) or positive affect spillover (*n* = 2) (Rodríguez-Carvajal et al., 2019; Stocker et al., 2014). Moreover, role theory-related approaches (*n* = 2) stemming from role conflict theory (Kahn et al., 1964) and boundary theory (Ashforth et al., 2000) were little used to explain negative and positive spillover processes. One paper did not specify a theory for spillover or recovery processes (e.g., Liu et al., 2021).

Regarding recovery processes, eight of the 21 papers included recovery-related constructs such as psychological detachment (e.g., Wang et al., 2019) or rumination (Syrek & Antoni, 2014) in their measurements. These studies applied process-based perspectives using the Effort Recovery theory (Meijman & Mulder, 1998) (*n* = 2), the stressor-detachment model (Sonnentag & Fritz, 2015) (*n* = 1), general recovery theory (Sonnentag et al., 2022), self-regulation theory (Barnes, 2012; Baumeister & Vohs, 2003) (*n* = 2), and stress-as-offense-to-self theory (Semmer et al., 2007) (*n* = 2) to include recovery processes in their EWB conceptualizations.

#### EWB theoretical approaches

Research on negative EWB (*n* = 10) is more recurring than positive EWB concepts (*n* = 7). Only four papers combined both negative and positive EWB indicators with respect to potential differences regarding positive and negative EWB processes.

Furthermore, research tends to focus more on affective wellbeing (*n* = 9) rather than on psychological wellbeing (*n* = 5) and seven papers studied a combination of affective and psychological wellbeing to account for the multifaceted nature of EWB. Research focusing on psychological wellbeing mainly uses work engagement as the most dominating positive indicator.

When affective elements were researched, studies used mainly negative indicators such as job-related depressive mood (Stocker et al., 2019) or anxiety (Cangiano et al., 2019), with very little research using positive indicators such as vitality (Rodríguez-Carvajal et al., 2019), happiness (Demerouti et al., 2013), or serenity (Stocker et al., 2014). The most recurring affective wellbeing indicator in work-nonwork interface research was emotional exhaustion (*n* = 5), another negative parameter.

Research focusing on job characteristics in forms of resources and demands was the most prevalent (*n* = 10), including conservation of resources theory (Hobfoll et al., 2018) (*n* = 4), job-demands resources theory (Demerouti et al., 2001) (*n* = 2), job demand control theory (Karasek, 1979) (*n* = 1), the Work-Home Resources model (ten Brummelhuis & Bakker, 2012) (*n* = 1), and the transactional stress theory (Lazarus & Folkman, 1984) (*n* = 1). In a similar way, a COVID-19 related paper applied event systems theory to focus on macro-contextual changes (Morgeson et al., 2015).

Aside from resource-demands perspectives, few motivational-related theories (*n* = 3) (Deci & Ryan, 1985) were applied. In two papers, self-determination theory was used to explain adverse effects of negative leadership on negative indicators such as ruminative thinking (Liang et al., 2018) and detachment (Cangiano et al., 2019). These examples suggest that abusive supervision impairs negative affective wellbeing, which results in negative spillover that impairs recovery processes. One paper (Rodríguez-Carvajal et al., 2019) used self-determination theory to highlight the beneficial effects of servant leadership on followers feeling of vitality through increased meaning in life throughout the day.

### Researched mechanisms in the leadership-EWB relationship

To answer the research question “What categories of mechanisms under study can be identified?”, we distinguish studies based on bolstering/hampering mechanisms (predictor function) and buffering/strengthening (moderator function) mechanisms. Perhaps unsurprisingly, the detected leadership-EWB mechanisms are complex. Research that used leadership as a predictor is most prevalent (*n* = 11) followed by nine studies that modelled leadership as a moderator in stressor/strain relationships. Mediation mechanisms are clearly under researched: Only one study modelled positive leadership as a mediator of a multi-level intervention-EWB relationship but failed to show significant mediation effects (Moen et al., 2016).

In general, bolstering/hampering mechanisms (i.e., predictor function) have been investigated more frequently in intervention studies and field experiments with longer time periods (*n* = 7/9 category 1), whereas buffering/strengthening (i.e., moderator function) mechanisms were investigated predominantly in more dynamic time frames with within-subject designs (*n* = 7/12 category 2).

#### Investigated bolstering/hampering mechanisms

The bolstering/hampering mechanisms were predominating (*n* = 12) and papers mostly investigated some sort of positive leadership and thus bolstering mechanisms (*n* = 9). Only three of them researched negative leadership, i.e., hampering mechanisms.

Category 1, which centers on the longer term studies, mainly followed the bolstering idea of leadership as a contextual macro-resource (*n* = 7) and used general leadership styles and forms of social support as well as more specific, nonetheless multidimensional, leadership behaviors. A clear distinction between relation-oriented and task-oriented forms of leadership support was not possible, since many papers theorized multiple pathways and included a mix of both forms of support in their measures. However, a reoccurring central argument for the bolstering effect of leadership was that leaders may positively influence employees’ work time flexibility (i.e., a resource) to either prevent negative spillover (Hornung et al., 2011; Munir et al., 2012;) or to boost positive spillover (Davis et al., 2015; Demerouti et al., 2013). Direct effects between positive leadership and EWB were inconclusive (Fan et al., 2019; Moen et al., 2016; Munir et al., 2012).

Category 2, which centers on the more dynamic studies (i.e., ESM studies), revealed more specific positive leadership behaviors such as appreciative leadership (Stocker et al., 2014) or upshifts in interpersonal justice behaviors (Wang et al., 2019) all focused on relationship-related leadership support containing elements of individual consideration. An example is the study of Stocker et al. (2014) showing that daily appreciation by the supervisor as a resource predicted positive affective wellbeing at the end of the workday, which was linked to important recovery processes such as energetic deactivation.

Regarding hampering mechanisms and the lesser researched negative leadership as a demand, only one longer term category 1 study showed that abusive supervision increased negative spillover and thereby negatively affected EWB. Specifically, somatic complaints elevated via increasing ruminative thinking off-work, suggesting that employees with abusive leaders fail to engage in needed recovery processes by replaying memories and prolonging detrimental social work experiences (Liang et al., 2018).

With regards to the more dynamic studies of category 2 (i.e., ESM studies), investigations of more specific negative leadership behaviors show that leadership can hamper EWB by affecting their sleep or recovery (Barnes et al., 2020; Wang et al., 2019). For example, Barnes et al. (2020) found that leaders who do not prioritize their workers' sleep, subsequently affect their sleep quality on a daily basis.

In sum, the evidence of how positive leadership can bolster dynamic EWB outweighs the evidence of how negative leadership hampers dynamic EWB.

#### Investigated buffering/strengthening mechanisms

As mentioned before, the buffering/strengthening mechanisms were less researched (*n* = 9) than bolstering/hampering mechanisms. Studies researching the role of positive leadership for EWB predominated (*n* = 6) and only three of them studied negative leadership.

Regarding positive leadership, studies applying ESM with a more dynamic perspective (category 2) (*n* = 4/6) were more frequent compared to only two studies that adopted a multiple wave design (category 1).

The buffering mechanism occurs via both task and relationship-oriented leadership support (Chong et al., 2020; Liu et al., 2015, 2021; Stocker et al., 2019). However, some findings are insignificant (Blanco-Donoso et al., 2017; Breevaart & Bakker, 2018).

As for less researched category 1 studies, the few available examples suggest that leadership as a resource can buffer stressor/strain relationships by communicating and increasing work meaningfulness (Liu et al., 2021) or by communicating appreciation (Stocker et al., 2019). For example, Stocker et al. (2019) showed in a two-wave study that the postulated job demand of job interruptions had no effect on job satisfaction, self-efficacy, job-related depressive mood, nor sleep problems when appreciation by supervisors was high, whereas these effects were significant when appreciation by the supervisor was low.

As for the predominating category 2 short term studies, two papers could show that positive leadership can buffer daily negative spillovers from work to nonwork (Chong et al., 2020), and vice versa (Liu et al., 2015). For example, Liu et al. (2015), could show that perceived managerial family support could buffer the effect of negative spillover from nonwork to work to emotional exhaustion later that day.

However, findings of this dynamic buffering effect of positive leadership are inconclusive, as some papers did not find the hypothesized relationships in this regard. E.g., Breevaart and Bakker (2018) could show on a sample with 271 elementary school teachers that the negative effect of daily role conflict on work engagement was only significant when daily transformational leadership was low (vs. high), but this moderating effect was not the case for daily family to work conflict on work engagement.

Regarding the few negative leadership studies (n = 3) following the strengthening mechanism, research suggests that negative leadership as a demand strengthens stressor/strain relationships via relationship-related negative leadership (Cangiano et al., 2019) and via negative role modelling leadership behavior (Derks et al., 2015; Syrek & Antoni, 2014).

**Fig. 2:**
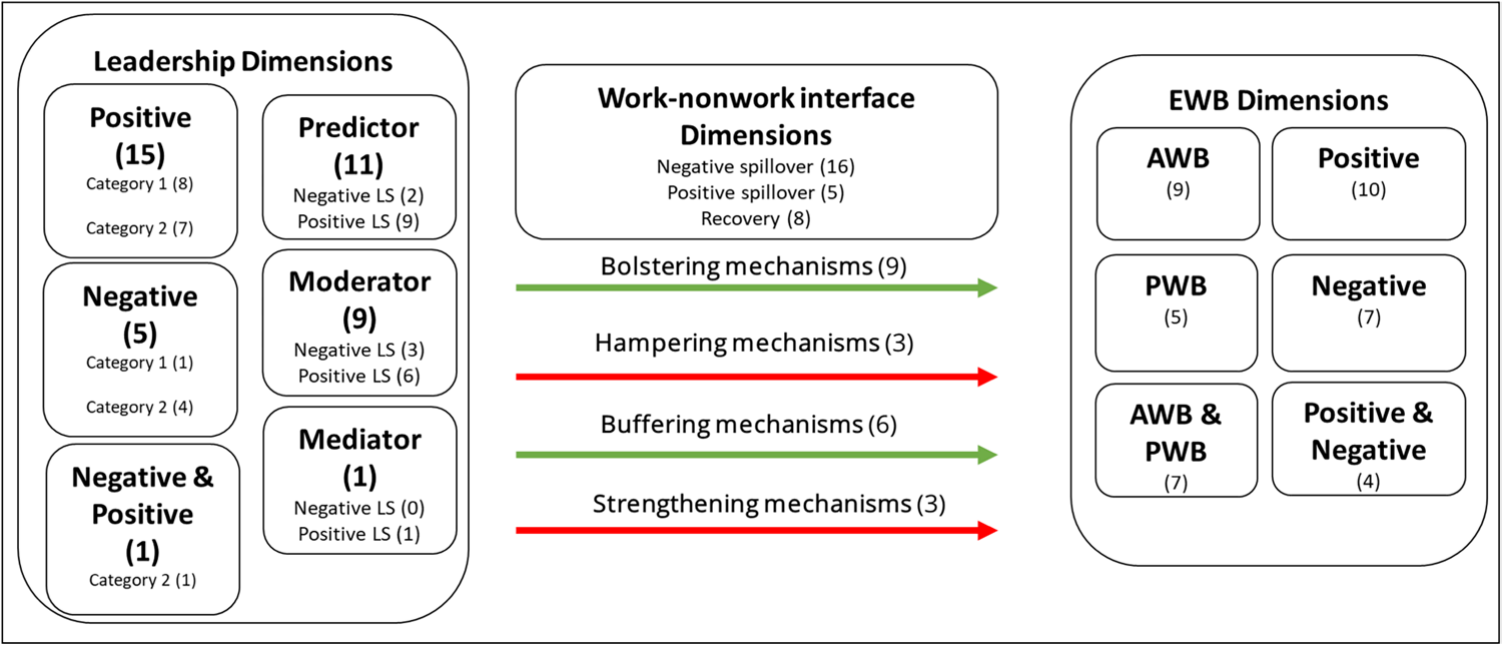
Overview of examined leadership, work-nonwork interface, EWB dimensions, and mechanisms in the reviewed papers. *Note*. LS = Leadership. EWB = Employee wellbeing. AWB = Affective wellbeing. PWB = Psychological wellbeing. Number in parentheses indicates number of identified papers

For example, Cangiano et al. (2019) found that high levels of punitive supervision accentuated the psychological risks of daily proactive behavior on negative EWB (i.e., anxiety), which was associated with less daily detachment after work the same day. As for negative role modelling behaviors, e.g., Derks et al. (2015) showed that negative leadership may increase stable contextual demands by focusing on the increased blurred boundaries intensified using internet and communication technologies. Hypothesizing that daily smartphone use in the evening hours is more strongly related to negative daily spillover for employees who are expected (vs. not) to stay online by their supervisor, they found indeed that an “always on”-culture set by the supervisor amplifies this detrimental relationship. Figure 2 provides an overview of examined leadership, work-nonwork interface, and EWB dimensions in the reviewed papers.

### Theoretical issues

To answer the research question “which theoretical issues can be identified?”, we observed several issues that are worth highlighting. Table 4 summarizes the identified theoretical and methodological issues.

**Table 4:** Theoretical and methodological issues identified in the screened articles

Theoretical issues	Methodological issues
***Issues regarding leadership approaches*** • Negative leadership is under-researched and limited to active direct and indirect forms • Social-relational perspectives limited to account for indirect mechanisms via working conditions. Work-home resource model and JD-R-R model under-researched • Leader’s indirect influence via shaping the psychosocial work environment conditions not sufficiently operationalized • Too much use of broad leadership and support styles • No support-spillover-fit/No differentiating between task-related and relationship-related support • Recovery processes are insufficiently addressed • Limited integration of the process perspective into the resource-demands perspective ***Issues regarding EWB approaches*** • Limited use of affect-driven theories and focus on affective energy • Positive psychology approach underdeveloped when focusing on spillover and recovery processes ***Issues regarding work-nonwork interface approaches*** • Positive spillovers are under-researched • Fine-grained distinction of differential spillover processes is insufficient ***Issues regarding researched mechanisms in the LS-EWB relationship*** • Lack of knowledge about hampering mechanisms. Task-related negative leadership under-researched • Insufficient use of job resources and demands as mediators • Influence of bolstering and hampering mechanisms for dynamic spillover and recovery processes unclear • Inconclusive findings of buffering mechanisms	Sampling: few heterogeneous samples, focus on working parents, predominance of IT and healthcare sector Dependency on company constraints Use of financial incentives Potential confounding effects: Multi-level interventions, use of compound scales

#### Issues regarding leadership approaches

We identified three main issues regarding theory development for leadership that are worth highlighting.

Firstly, research is unbalanced and focuses predominantly on positive leadership as a resource. Negative leadership is under-researched and limited to active direct and indirect forms. Moreover, the influence of passive forms of negative leadership, i.e., its absence in form of laissez-faire or passive-avoidant leadership, on spillover, recovery, and EWB has not been researched yet longitudinally.

Secondly, we observed that social-relational frameworks such as social exchange or social learning theory that focus predominantly on the dyadic leader–follower interaction might be limited to explain indirect mechanisms via working conditions on spillover and recovery processes. To include specific resources and demands, rather the resource-demands perspective and related specific frameworks that account for the work-nonwork interface seem more suitable. However, the work-home resource model has only been used scarcely. Most notably, the job-demands-resources-recovery model (Kinnunen et al., 2011) has not been used in any of the investigated longitudinal papers, although it has recently been successfully applied in cross-sectional research (Dolce et al., 2020). Thus, we consider the limited use of mediators, and the limited integration of the process perspective into the resource-demands perspective a theoretical gap that warrants more process-based theory development and empirical testing.

Ultimately, the diverse number of applied leadership constructs, ranging in their breadth from broad styles to general and more specific forms of support, seem to complicate clear evidence synthesis. In other words, the use of broad multidimensional or general supervisor support constructs identified in some of the present studies are limited in their explanation regarding which specific leader behavior directly or indirectly affects spillover, recovery processes and EWB. More specific behaviors such as family-supportive supervisor behavior remain scarce, and single facets of broader concepts are also seldom used. The distinction of task- or relationship-oriented support is widely missing.

#### Issues regarding EWB approaches

As expected, the literature scoping review confirmed that the conceptualizations of EWB are too narrow and need to be expanded, and that recovery processes have been insufficiently addressed. In this regard, we detected two main theoretical gaps, which refer to the limited use of affect-driven theories and the lack of positive psychology approaches.

Specifically, while affect and affective spillover across the work-nonwork interface was the focal construct of many of the identified articles, only two papers (Stocker et al., 2014; Wang et al., 2019) included affect-driven theories such as the circumplex model of affect (Russell, 1980). For example, Wang et al.’s (2019) study used this theory to emphasize the importance of psychological energy activation for work and deactivation for recovery. This differs from broader resource-demands based theories that do not allow this nuanced view on different forms of personal energy. The limited use of such theories is problematic, as the role of affective energy, prolonged affective activation, and deactivation could be identified as a central theme in many of the reviewed studies.

Furthermore, motivational theories such as self-determination theory and linked psychological wellbeing constructs seem under-researched. In general, the positive psychology approach seems underdeveloped when focusing on spillover and recovery processes, while reducing work-nonwork conflict and associated emotional exhaustion seem to be of predominant relevance. In other words, whereas the buffering idea suggests that leaders might break negative spirals, not much is known about how leaders can onset positive spirals. Research building on prominent resource-based positive psychology theories such as the prominent broaden-and-build theory (Fredrickson, 2004) is missing here, which could shed light on the positive linkages between resources that need optimizing. This view is also echoed in conservation of resources theory under the term of “resource caravan passageways” (Hobfoll et al., 2018, p. 107), and might be particularly interesting for a more resource-based approach for investigating spillover and recovery processes in the leadership-EWB relationship.

#### Issues regarding work-nonwork interface approaches

Our scoping review has revealed that negative conflict-oriented studies are predominant, while positive spillovers are vastly under-researched. Thus, positive spillover needs further investigation and leadership-EWB research has to be more dynamic if the above-mentioned positive spirals are to be properly identified. Moreover, a more fine-grained distinction of spillover processes is warranted, as leaders might influence different forms of spillovers in different task- or relationship-oriented supportive ways. For example, spillover processes may be based on time and energy resources (Geurts et al., 2005), or may be affective-based, instrumental-based, and value-based (Hanson et al., 2006), and a stronger alignment between specific leadership influence and specific spillover might result in stronger effects. Although the limited number of articles prevents us from drawing a robust picture of this hypothesized support-spillover-fit, the present research mapping indicates, e.g., that positive leadership can, on the one hand, preserve and increase time resources by providing task-related supportive leadership (e.g., in the form of increased work scheduling autonomy), and, on the other hand, preserve and increase energy resources through relationship-related support (e.g., appreciative behaviors, increase of experienced energy levels at work).

#### Issues regarding researched mechanisms in the leadership-EWB relationship

We identified some theoretical gaps regarding the mechanisms that have been researched. Generally, the limited total number of articles highlights the need for more longitudinal research in this area. Furthermore, evidence of how positive leadership can bolster EWB by optimizing the work-nonwork interface of their followers outweighs the evidence of how negative leadership hampers dynamic EWB; the latter needs more investigation. The few available studies suggest that negative leadership hampers recovery and EWB by role modelling adverse behaviors (sleep devaluation, unhealthy performance expectations) or through perceived interpersonal injustice which depletes personal energy resources and undermines recovery processes. However, not much is known about task-related negative leadership that would increase job demands. We also observed that many of the reviewed studies only theorized the influence of leadership on job resources and demands but did not include them as mediators and rather investigated more simple relations. Linked to this, although often theorized as a daily variable (for a related review see Kelemen et al., 2020), research has suggested both empirically (Breevaart & Zacher, 2019) and theoretically (Bakker & de Vries, 2021), that leadership may best be conceptualized as a rather stable contextual macro resource, or macro demand, respectively. As such, it has been suggested that a large portion of leadership’s influence might indeed be exerted more indirectly through the leader’s prominent agent role in shaping the psychosocial work environment conditions, influencing both other job resources and job demands (e.g., Berger et al., 2019; Schaufeli, 2015). Notably, boundary conditions for leadership apply here, depending on the leader’s role capacity to change working conditions (Bakker & de Vries, 2021). For example, the reviewed rigorous multi-level intervention study by Moen et al. (2016) showed that increased work schedule control significantly reduced negative spillover and increased wellbeing, but that the changes in leadership behavior alone did not have this desired effect.

Additionally, both bolstering and hampering mechanisms need more dynamic investigations in the form of experience sampling method studies to address leader’s influence on spillover and recovery processes via fluctuating demands and resources. Ultimately, buffering mechanisms are inconclusive, which is why more coherent theory-based investigations are needed.

### Methodological issues

Before answering the research question “Which methodological issues and potential remedies can be identified?”, it is worth noting that data quality of most of the retrieved studies was high, strengthening the importance of applying more rigorous research methods rather than cross-sectional designs. The GRADE rating resulted in almost perfect inter-rater agreement (Cohen's κ = 0.809) (Landis & Koch, 1977). Most of the studies yielded a high-quality rating (67%; see Annex Table 6). Nonetheless, we detected some methodological issues and potential remedies surrounding the design, results, and external validity of the identified studies that are worth highlighting in order to guide future research. Note that this does not imply any general devaluation of any of the studies in question.

Regarding the design and external validity, we identified issues relating to the sampling and temporal order of the variables. That is, many studies centered their sampling around specific organizations (e.g., Danish elderly care organization; Munir et al., 2012) and sectors (e.g., IT sector; Fan et al., 2019), whereas other studies used a largely selective sample (e.g., only working parents in Japan with children under the age of six; Demerouti et al., 2013). Only a few studies applied heterogeneous samples which might be more representative (Cangiano et al., 2019; Derks et al., 2015; Stocker et al., 2014). Although sample specification can produce more robust evidence within the population under study (Barnes et al., 2020), it limits the generalizability of these studies to the wider population (Demerouti et al., 2013). Additionally, we found that most of the evidence stems from the IT sector (*n* = 5) or the medical staff sector (*n* = 4).

Related to this, company collaborations were an issue, as it requires considerable number of financial resources and makes research considerations dependent on company requests and practices rather than theoretical reasoning. For example, Liu et al. (2021) highlighted that their intervention study had to be switched from an initially planned 3-weeks timeframe to a 2-weeks timeframe as per the investigated hospital’s request. Another issue may be that more sensitive topics may not be feasible to investigate as per company constraints. It is noteworthy that all studies that addressed negative leadership mobilized personal networks for sampling instead of engaging in company collaborations. Almost all of the category 1 studies and few category 2 studies collaborated with specific companies to recruit their samples. However, it is important to note that close contact with HR departments during the design of the study may also be beneficial for several reasons. Firstly, possible leadership behaviors can be more accurately specified to the target population under study (e.g., Breevaart & Bakker, 2018), which is important for assessing potential reach of influence of the leader. Secondly, whereas time horizons might be shortened due to company constraints, the timepoints for daily data collection might be defined more accurately based on the actual working hours of the participating employees (e.g., Liu et al., 2015). Finally, especially for category 2 studies, company collaborations might make it easier to reach sufficient sample sizes, which is often an issue for intensive studies (Gabriel et al., 2019). The other nine ESM studies mobilized personal networks and broader university alumni networks to find suitable study participants.

Moreover, regarding financial incentives and external validity, in four cases, financial incentives were offered for participation. Although financial incentives may be particularly useful for raising the number of participants for ESM studies, such incentives may unintentionally affect data quality through the rise of arbitrary response options to increase participant eligibility or unequal attractiveness of the incentive for different potential participant segments (Gabriel et al., 2019).

As for the results, some studies of category 1 reported potential confounding effects. That is, in some cases, leadership behavioral changes and its effects on EWB were part of multi-level interventions and its effects were not decomposed. In other words, it was not clear if the change in perceived leadership behavior or other actions around the intervention affected spillover, recovery, and EWB (e.g., Davis et al., 2015; Moen et al., 2016). In another case, report of p-values of significance was missing (Fan et al., 2019). Moreover, two studies applied measures that were not exclusively addressing leadership behavior. Specifically, Breevaart and Tims (2019) used a compound scale to measure social support from both colleagues and supervisors, and Chong et al. (2020) examined a construct called “telework task support”, which should perhaps be conceptualized as an organization-wide resource.

In line with the methodological issues mentioned above, some potential remedies can be identified. To ease control of data quality, it was argued that the use of time-sampling might be more useful than event-sampling (Stocker et al., 2014), and electronic designs might outplay paper–pencil designs (Liu et al., 2015; Rodríguez-Carvajal et al., 2019; Stocker et al., 2014). Also, as there are many jobs where leadership interactions might not occur daily, leadership perceptions may be measured once in a baseline survey instead of including it in daily surveys (Barnes et al., 2020). Generally, frequency of interactions with supervisors should be controlled for in the sampling and/or analysis process (Liang et al., 2018; Rodríguez-Carvajal et al., 2019). Another important aspect is to assess focal variables at different timepoints to reduce the risk of inflating relationships based on mood-dependent memory and to generally overcome the endogeneity problem. For example, Derks et al. (2015) assessed all variables at one measurement point, that is, at the end of a workday. Also, testing recovery processes at the end of the day, at a time when the recovery process itself should be taking place (e.g., Blanco-Donoso et al., 2017), might unintentionally affect the recovery experience itself and thereby data quality (Bolger & Laurenceau, 2013).

## Discussion

Our scoping review contributes to the longitudinal leadership-EWB literature by providing a helpful overview of approaches, concepts, and mechanisms across the work-nonwork interface as well as over theoretical and methodological trends and issues to inform future research. We identified an increase in research interest on this topic over the past four years. In the following, we want to offer suggestions based on our findings to advance the field both theoretically and methodologically (Table 5).

**Table 5:** Theoretical and methodological suggestions for future research

Advancing theoretically	Advancing methodologically
• Integrate multiple research streams (leadership, work-nonwork, EWB research • Instead of relying on established “positive” leadership concepts that might have double-edged effects for spillover and recovery processes, focus on recovery-supportive leadership behaviors • Focus on personal energy resources • Focus more on affective-driven theories such as the circumplex model of affect to address the multi-faceted concept of EWB and shifts in affective energy resources • Investigate the role of passive or absent leadership • Distinguish between task-related and relationship-related support • Focus on bolstering mechanisms to detect other resources than work scheduling autonomy • Apply an expanded leadership-JD-R-R model to frame leadership as macro-resource or demand • Explore leaders influence on stable resources/demands and the interaction of stable and dynamic resources and demands (e.g. moderated moderations)	• Use the experience sampling method • Be aware of potential pitfalls when engaging in company collaborations (atheoretical temporal lenses, limits of generalizability) • Be aware of potential pitfalls when providing financial incentives and explore immaterial incentives • Use more inclusive samples beyond working parents and address other sectors than IT and healthcare • Model multilevel: Leadership behaviors or styles as an upper-level predictor or moderator to operationalize the idea of the leader as a contextual macro resource • Use theory-based temporal lenses • Specify the instruments to assess leadership behavior (task-related or relational-related support) to avoid potential confounding effects • Embrace methodological complexity rather than aiming at simple relations

### Advancing theoretically

We would like to offer some recommendations to advance the longitudinal leadership-EWB research theoretically.

To begin with, at this developmental stage of the research field, our findings reveal a clear need to integrate multiple research streams (leadership, work-nonwork, EWB research) to fully grasp the intricacies of the leadership-EWB relationship. In line with Inceoglu et al. (2018), we therefore argue that embracing a dynamic resource-demand-based process perspective with theoretical complexity will result in a deeper understanding of how leaders influence sustainable EWB (Hofmans et al., 2021). As digitalization and flexibilization transformations will likely continue to blur the boundaries between work and nonwork and challenge vital recovery processes (Sonnentag et al., 2022), the way forward is to integrate work nonwork research into leadership-EWB research. Leadership can promote sustainable EWB (Di Fabio, 2017) only if spillover and recovery processes are adequately addressed. Related models such as the work-home resource model (ten Brummelhuis & Bakker, 2012) or the job-demands-resources-recovery model (Kinnunen et al., 2011) warrant more exploration for leadership.

Here, hitherto imagined “positive” leadership styles that are in essence performance-driven, need reevaluation, particularly since direct effects between positive leadership and EWB were inconclusive longitudinally (Fan et al., 2019; Moen et al., 2016; Munir et al., 2012). As such, we believe that merely approaching this topic from a broader “positive” leadership theory standpoint (e.g., transformational leadership) – which largely adopts a leadership effectiveness point of view – does not sufficiently explain the complex spillover and recovery mechanisms at play (Inceoglu et al., 2018). For example, while transformational leadership has been referred to as “energizer” (Schippers & Hogenes, 2011, p. 195), if the primary focus is on optimizing effort, but not on recovery, then this leadership style could overtax their followers’ energy system and thereby detrimentally impact recovery processes (Quinn et al., 2012; Syrek & Antoni, 2014). The reviewed papers that linked positive leadership types to work engagement (Breevaart & Bakker, 2018; Hornung et al., 2011) and challenging demands (Breevaart & Bakker, 2018), and high-performance expectations that impair recovery (Derks et al., 2015; Syrek & Antoni, 2014), support this claim. Thus, instead of relying on established broad “positive” leadership concepts that might have double-edged effects for spillover and recovery processes, we call for more research on more specific recovery-supportive leadership behaviors, which, e.g., support positive spillover processes and adequately balance employee’s energy resources (Crain et al., 2018; Parker et al., 2021; Quinn et al., 2012).

In line with this, when focusing on employee’s energy resources, we call for more precise conceptualizations of EWB and to investigate potential trade-offs between affective and psychological wellbeing effects of leadership (Taris & Schaufeli, 2015). To do so, our findings suggest that theory could be advanced by focusing more on affective-driven theories such as the circumplex model of affect (Russell, 1980) for several reasons. Firstly, the identified research papers that used this model (Stocker et al., 2019; Wang et al., 2019) could address spillover and recovery processes better by addressing employee’s affective energy resources. Additionally, the model includes many facets of both affective and psychological wellbeing elements such as stress (= high negative arousal), motivation (= high positive arousal), serenity/relaxation (low positive arousal), or fatigue/exhaustion (= low negative arousal), and thereby allows for an improved definition of the favorable versus unfavorable processes. For example, in the short term, i.e., on a daily level, we might refer to optimal states of moderate to high active positive affect in the work domain (feeling energized, enthusiastic) and optimal states of moderate to low active positive affect in the nonwork domain (feeling serene, at ease). This process perspective integrates affect-based theories (Weiss & Cropanzano, 1996) with recovery theories (Meijman & Mulder, 1998) and facilitates the testing of unfavorable trade-offs between, e.g., energizing, and motivational effects of positive leadership during work on the one hand and, on the other hand, feeling relaxed and calm off-work. It also includes cognitive and biochemical elements and thereby integrates neuroscience into I/O psychology (e.g., Posner et al., 2005). This supports the use of more objective and physical data collections, that have been called for by some of the reviewed studies (Barnes et al., 2020; Moen et al., 2016; Liang et al., 2018; Stocker et al., 2014).

Additionally, we recommend applying a distinction between task- or relationship-oriented support (Yao et al., 2021). This distinction has been widely missing in the reviewed papers. Following this idea, researchers could differentiate better between bolstering/hampering versus buffering/strengthening mechanisms, and accordingly adopt differential views of leaders as preventers or interveners. Regarding the latter, our review has shown that while leadership is mostly investigated as a resource, spillover and recovery processes have mostly been addressed from a demands-based perspective. The related studies on buffering effects could show that leaders as resources can buffer stressor/strain relationships and thereby intervene in negative spillover and impaired recovery processes (Chong et al., 2020; Liu et al., 2015, 2021; Stocker et al., 2019). By applying the distinction here, relationship-related leadership support such as communicating appreciation or work meaningfulness could be more important for buffering, i.e., coping, and emotional regulation processes. Accordingly, specific relationship-related leadership support behaviors could be tested as moderating functions of stressor-strain mechanisms that spillover in the nonwork domain.

**Fig. 3:**
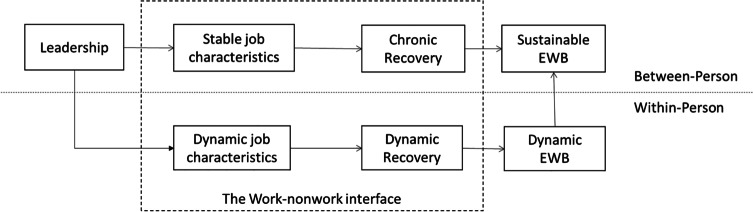
The proposed bolstering/hampering mechanisms in the leadership-JD-R-R model

However, a positive psychology approach with a stronger focus on resources, positive spillover and recovery processes is needed to increase the understanding of leaders as preventers rather than interveners. As our findings suggest, leaders may bolster instrumental resources such as scheduling autonomy by managing workload, but less is known about how other contextual resources affect spillover and recovery processes positively. For example, leaders may also grant more decision authority beyond scheduling autonomy (i.e., regarding where and how they work) to their followers (Fan et al., 2019). Moreover, leaders might increase personal development (e.g., Hornung et al., 2011) as well as personal energy resources (Breevaart & Bakker, 2018) and thereby positive work experiences (Fredrickson, 2004) that facilitate positive spillover and recovery processes. Therefore, regarding more preventive rather than intervening research following bolstering/hampering mechanisms, we recommend theorizing leadership as a stand-alone factor that influences job characteristics. This is also in line with previous research (Berger et al., 2019; Schaufeli, 2015). To theoretically frame the contextual influence of leadership within these processes, an expanded leadership-JD-R-R model may offer the most suitable approach, as it represents a resource-demands-based process perspective and as such transcends relational theories. In this regard, we suggest applying a between-person view for leadership constructs and stable job characteristics, and a within-person person view for more dynamic job characteristics (see Fig. 3).

Moreover, as our review has shown, more research is warranted to investigate the influence of passive forms of negative leadership on employees’ spillover and recovery processes and EWB. Although none of the reviewed papers investigated this form of leadership, previous research has shown that passive forms of negative leadership are more prevalent than active forms (e.g., Aasland et al., 2010). Also, recent cross-sectional research suggests that it might be particularly harmful in remote work conditions (e.g., Wang et al., 2021). Passive leadership has been related to higher levels of job demands (Berger et al., 2019), and high level of job demands result in negative spillover and impaired recovery processes (e.g., Blanco-Donoso et al., 2017; Moen et al., 2016; Syrek & Antoni, 2014). It is therefore important for EWB preventive measures to increase knowledge about this particularly negative leadership style.

**Fig. 4:**
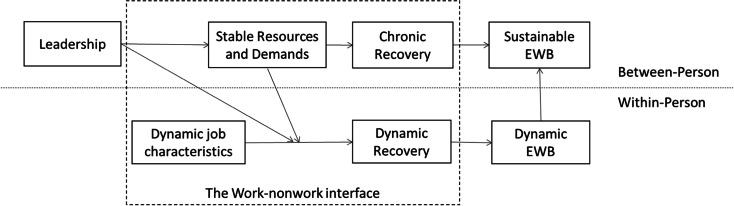
The proposed buffering/strengthening mechanisms in the leadership-JD-R-R model

For research interested in investigating buffering/strengthening mechanisms, we recommend exploring differential moderating functions of leadership on prevalent job demand-strain functions (see Fig. 4). Additionally, although not researched in the reviewed in papers, leaders might influence not only dynamic personal resources such as energy but also more stable key personal resources (e.g., optimism) that in turn influence job demand-strain functions (ten Brummelhuis & Bakker, 2012), and thereby act as a higher-order moderator. In this sense, personal characteristics of the employees might moderate the investigated mechanisms. Our review evidences that this interaction of stable and dynamic resources and demands at personal and contextual level warrants more investigation. Leaders may, for example, influence rather stable work conditions (e.g., by regulating individual decision authority, by setting norms for performance expectations, by establishing an “always on”-culture, etc.) which, in turn, interact either positively or negatively with more dynamic job characteristics (e.g., daily workload, daily scheduling autonomy) that are affecting spillover and recovery processes (Bakker & de Vries, 2021). Here again, we call for more fine-grained research that distinguishes theoretically between specific leadership support types (e.g., task- or relationship-related) rather than broad positive versus negative leadership styles and better theoretical alignment between these behaviors with different types of spillovers across the work-nonwork interface (e.g., affective versus instrumental spillover; Hanson et al., 2006).

Finally, we encourage researchers to potentially expand the models presented here. For example, leaders' personal characteristics as well as their own contextual working conditions might also influence their exerted leadership (Berger et al., 2019), provoke so-called crossover effects, and thereby influence employee’s work-nonwork interface (Nielsen & Taris, 2019). For example, Sonnentag and Schiffner’s cross-sectional study (2018) revealed that leader recovery was related to employee recovery. They suggested that a shared environment, stressful or not stressful, might evoke the same reactions in two individuals. In turn, crossover effects from employee to leader that influence spillover and recovery processes are also possible (Nielsen & Taris, 2019), thus suggesting a more bidirectional rather than unidirectional leadership-EWB relationship. Future research may want to further explore and test these ideas in diary leader–follower dyads designs.

### Advancing methodologically

Generally, the strength of the experience sampling method to assess life as it is lived becomes apparent. Accordingly, we call for more experience sampling method studies for this research field. Our review shows that diary studies are better suited to capture spillover and recovery processes, whereas longitudinal studies with longer time frames may be more suited for broader intervention studies but limited in addressing these dynamics. The most common occupational health theories including stressor/strain theories, regulation and affective theories imply a shorter dynamic temporal lens and within-person effects (Cham et al., 2021; Kelemen et al., 2020; Sonnentag et al., 2022). Yet, our identified methodological issues show that these study designs are complex and thus require careful consideration. For example, researchers should weigh the potential advantages of company collaborations regarding sample size and specification as well as knowledge about leaders’ potential range of influence with the potential downsides of rather arbitrary and atheoretical temporal lenses and limits of generalizability. Electronic, time-sampling designs can assure that data entries fit the theorized lens. Also, rather than providing financial incentives for participation, immaterial incentives such as the provision of individual feedback may be explored to reduce the risk impoverished data quality (Gabriel et al., 2019). In any case, potential self-selection bias should be considered. As our review shows that most knowledge relates to work-family spillover and stems from the IT and healthcare sector, future research should use more inclusive samples beyond the ones typical for work-nonwork research (i.e., working parents with young children) and address other sectors.

Experience sampling methods seem to facilitate the alignment of theoretical complexity with feasible methods: modelling leadership behaviors or styles as an upper-level predictor or moderator in demand- or resource-based EWB processes in dynamic multi-level models seems the most appropriate modelling method to operationalize the idea of the leader as a contextual macro resource. We call for more inclusive research that selects theory-based temporal lenses. For example, regarding bolstering/hampering mechanisms, specific or broader leadership behaviors may be measured temporally prior to the dynamic assessments to align method with theory. In contrast, regarding buffering/strengthening mechanisms, specific leadership behaviors may be assessed at the daily level. To avoid potential confounding effects, research should clearly specify the instruments to assess leadership behavior to rule out other social support, e.g., by co-workers, or other contextual effect changes. Clearly distinguishing between task-related and relation-related forms of support and investigating its differential effects would increase theory-method fit. Finally, we encourage future scholars to embrace methodological complexity and include the interaction of resources and demands rather than investigating only simple relations and aiming at the most parsimonious models (Hofmans et al., 2021) to increase theory-method fit (Vantilborgh et al., 2018). Methodological remedies and recommendations are available (see Bolger & Laurenceau, 2013; Ohly & Gochmann, 2017) and advancing (e.g., Gabriel et al., 2019), and this scoping review suggests that even studies with limited small sample sizes (e.g., Wang et al., 2019) can contribute significantly to the existing research body through presenting methodological rigor and interesting methods.

### Practical implications

Our study has revealed several practical implications. Firstly, with regards to EWB, we have demonstrated the importance of focusing on spillover and recovery processes for policy-makers and managers alike is demonstrated. Leaders should be aware that personal energy is finite and its short-term restoration crucial to sustain EWB. Secondly, it is evident that diverse leadership styles influence underlying processes differently. To facilitate recovery processes, leaders – or organizations – may provide instrumental support to their followers in the form of more decision authority relating to when, where, and how they work. To cope with demands, leaders should provide relationship-related support via e.g., communicating meaningfulness or appreciation. Leaders should be aware that high performance expectations and energizing behaviors may backfire if the importance of necessary recovery-related boundaries are not communicated adequately. Negative leadership that undermines recovery processes by devaluing sleep or other related negative role modelling behaviors impair EWB. Also, leaders should be aware that the recovery processes of followers are heavily influenced fearing punitive actions or experiencing unjust treatment. For policymaking around EWB and leadership training and development, it is thus pivotal to address the work-nonwork interface and recovery-supportive leadership behaviors.

## Limitations

As with other reviews, our current study has several limitations. Firstly, we examined only empirical published manuscripts. This limitation did not allow us to analyze unpublished studies from scholars and investigations presented at conferences. Secondly, we acknowledge that, although we applied the PRISMA-ScR and a transparent literature search strategy, other articles might relate to our research goal that we did not detect. This may be so because the concepts of EWB and work-nonwork interface are not yet sufficiently linked, which makes it difficult to clearly identify articles. For example, Kelemen et al.’s review (2020) on daily LS found 8 out of 74 articles before 2011, whereas we did not identify any of these studies as relevant for our research that focused particularly on spillover and recovery processes. However, Munn et al. (2018) stated that scoping reviews are particularly useful ‘when clarification around a concept or theory is required’ (p. 5), and our results aimed at targeting theory expansion and clarification of its complexity. Finally, this scoping review was an enormous undertaking, and our results are only up to date as of February 2021.

## Conclusion

This scoping review is the first to organize the longitudinal evidence of an emerging research topic, that is, the role of spillover and recovery processes in the leadership-EWB relationship. The evolution towards increasingly blurred boundaries between work and nonwork and thus increased relevance of the work-nonwork interface has been insufficiently addressed by the leadership literature. Scoping the existing literature through an integrative resource-demands-based process perspective allowed for the much-needed expansion of the existing leadership-EWB relationship. To this end, we proceed to identify the various theoretical approaches and map evidence of two main mechanisms, i.e. bolstering/hampering mechanisms versus buffering/strengthening mechanisms, and highlight theoretical and methodological issues. In doing so, we hope to spur future exploration of the topic and redirect future research towards the most promising theoretical and methodological instruments, while providing practitioners and policy-makers with ideas about how to address spillover and recovery processes in order to sustain EWB.

## Data Availability

Data sharing not applicable to this article as no datasets were generated or analysed during the current study.

## Appendix

### Complete list of search terms used for the literature search in our review

#### Leadership search terms (based on Montano et al, 2017)

Leadership* OR "Middle Level Managers" OR "Supervisor Employee Interaction" OR "Manager Employee Interaction" OR "Employee Supervisor Interaction" OR ((leader* OR supervisor*) N1 (qualit* OR behavio* OR style* OR skill* OR characteristic* OR traits OR attributes OR personality OR attitude* OR abus* OR destructive OR aggressi* OR negative OR tyrannic* OR undermining OR psychopathic OR toxic OR despotic OR "laissez faire" OR passive OR narcissistic OR transform* OR transact* OR charisma* OR "health-specific" OR "health-domain" OR "health-oriented" OR authentic OR ethic* OR shared OR servant OR distributed OR collective OR collaborative OR consensus OR climate)) OR "consideration and initiating structure" OR "petty tyranny" OR bossing OR "leader-member-exchange" OR LMX OR "leader-following consensus" OR "leader–follower agreement" OR "leader-member agreement" OR "group level leader*") NOT (PO Animals).

#### Wellbeing search terms (based on Montano et al., 2017)

("mental health" OR "psychological health" OR "well-being" OR wellbeing OR "job related affective well-being" OR eudaimonia OR "quality of life" OR "quality of work life" OR "quality of working life" OR "quality of worklife" OR "work ability" OR "working ability" OR workability OR "performance capability" OR "capability of performance" OR employability OR "evidence of functioning" OR "social functioning" OR "psychological functioning" OR flourishing OR "meaning in life" OR "purpose of life" OR "mental prosperousness" OR prosperousness OR "health effects" OR "health implications" OR "impairment to health" OR "health detriment" OR "health consequences" OR "health 4 status" OR "state of health" OR "health situation" OR "health disorders" OR "health hazard" OR "health risk" OR "health problem" OR "health issue" OR "danger to health" OR "benefit to health" OR "health benefit" OR "emotional state*" OR "positive emotion*" OR "positive feeling*" OR "positive affect*" OR "workplace emotion*" OR "negative affect*" OR "negative emotion" OR "negative feeling*" OR positivity OR "affect balance" OR mood OR moods OR "emotion expressiveness" OR "expressive emotion" OR gladness OR happiness OR "mental balance" OR vitality OR vigilance OR sadness OR "Boredom" OR "psychologic* stress" OR "psychological strain*" OR "psychological distress*" OR "chronic stress*"OR "distress" OR "job stress" OR "job tension" OR "job-induced tension" OR "job strain" OR "work stress" OR "job related strain" OR "job related stress" OR "work related stress" OR "occupational stress*" OR "workplace stress" OR "organizational stress*" OR "organisational stress*" OR "Stress Reactions" OR ((employee* OR subordinate* OR follower*) AND (stress* OR strain OR coping)) OR fatigue OR lassitude OR "general tiredness" OR "Sleepiness" OR sleep quality OR exhaustion OR exhausted OR nervousness OR irritation OR irritability OR anxiety OR frustration OR agitation OR hypervigilance OR "rumination (cognitive process)" OR cogitation OR "need for recovery" OR (((complain* OR symptom* N1 (health OR psycholog* OR psychosomati* OR psychovegetative* OR psychophysiolog*) OR "psychosomatic disorder" OR "somatoform disorders" OR tinnitus OR headache* OR "unspecific symptoms" OR "nonspecific symptoms" OR "unexplained symptoms" OR "unspecific pain" OR "nonspecific pain" OR "unexplained pain" OR "unspecific complaints" OR "nonspecific complaints" OR "unexplained complaints" OR discomfort OR "chronic pain" OR "chronic complaints")).

#### Longitudinal search terms

Longitudinal OR panel OR diary OR daily*

#### WNWI search terms used for screening the identified articles (based on Beigi et al., 2019)

“Work-social system adaptation” OR “Work-family accommodation” OR “Work-family boundary” OR “Work-social system fit” OR “Work-family balance” OR “Work-family conflict” OR “Work-family articulation” OR “Work-family border” OR “Work-family enrichment” OR “Work-family combination” OR “Work-family congruence” OR “Work-family facilitation” OR “Work-family harmony” OR “Work-family compensation” OR “Work-family spillover (positive)” OR “Work-family interaction” OR “Work-family enhancement” OR “Work-home interaction” OR “Work-family interface” OR “Work-family expansion” OR “Work-family intersection” OR “Work-family integration” OR “Work-family linkage” OR “Work-family fit” OR “Work-family management” OR “Work-family resource drain” OR “Work-leisure compensation” OR “Work-family segmentation” OR “Work-leisure segmentation” OR “Work-family spillover” OR “Work-leisure spillover” OR “Work-home conflict” OR “Work-home segmentation” OR “Work–nonwork conflict” OR “Work-life balance” OR “Work/nonwork expansion” OR “Work-life harmony” OR “Work-nonwork enhancement” OR “Work-nonwork compensation” OR “Work/nonwork segmentation” OR “Work-nonwork integration” OR “Work-nonwork spillover OR “Work-home enrichment” OR “Work/nonwork interface” OR “Work-nonwork enhancement” OR “Work–home interface” OR “Work nonwork intersection.

## Annex

Table 6

**Table 6:** Adapted GRADE rating of the screened articles

Index-Nr	Category	Reference (Year)	Sample recruitment	GRADE rating	Reasons for downgrading
1	Category 1	Davis et al. (2015)	Company collaboration	Moderate ***	Potential confounding effects (Effects of intervention actions were not tested independently)
2		Demerouti et al. (2013)	Company collaboration	High ****	None
3		Fan et al. (2019)	Company collaboration	Low **	Low effect sizes and missing sig. levels
4		Hornung et al. (2011)	Company collaboration	High ****	None
5		Liang et al. (2018)	Financial remuneration	High ****	None
6		Liu et al. (2021)	Company collaboration	Moderate ***	Arbitrary temporal lense
7		Moen et al. (2016)	Company collaboration	Moderate ***	Relationship is not significant
8		Munir et al. (2012)	Company collaboration	Low **	Mediator is not assessed time-lagged
9		Stocker et al. (2019)	Company collaboration	High ****	None
10	Category 2	Barnes et al. (2020)	Financial remuneration	High ****	None
11		Blanco-Donoso et al. (2017)	Private network	High ****	None
12		Breevaart and Bakker (2018)	Company collaboration	High ****	None
13		Breevaart and Tims (2019)	Company collaboration	Low **	Potential confounding effects (Constructs of Supervisor vs. Colleague as social resources were not investigated independently)
14		Cangiano et al. (2019)	Private network	High ****	None
15		Chong et al. (2020)	Financial remuneration	Low **	Potential confounding effects (Items address "organizational support" rather than specifically direct supervisor support)
16		Derks et al. (2015)	Private network	High ****	None
17		Liu et al. (2015)	Company collaboration	High ****	None
18		Rodríguez-Carvajal et al. (2019)	Private network	High ****	None
19		Stocker et al. (2014)	Private network	High ****	None
20		Syrek and Antoni (2014)	Private network	High ****	None
21		Wang et al. (2019)	Financial remuneration	High ****	None
